# Involving End Users in Adapting a Spanish Version of a Web-Based Mental Health Clinic for Young People in Colombia: Exploratory Study Using Participatory Design Methodologies

**DOI:** 10.2196/15914

**Published:** 2020-02-06

**Authors:** Laura Ospina-Pinillos, Tracey A Davenport, Alvaro Andres Navarro-Mancilla, Vanessa Wan Sze Cheng, Andrés Camilo Cardozo Alarcón, Andres M Rangel, German Eduardo Rueda-Jaimes, Carlos Gomez-Restrepo, Ian B Hickie

**Affiliations:** 1 Brain and Mind Centre, Sydney The University of Sydney Sydney Australia; 2 Department of Psychiatry and Mental Health Faculty of Medicine Pontificia Universidad Javeriana Bogotá Colombia; 3 Neuropsychiatry Research Group Universidad Autonoma de Bucaramanga Bucaramanga Colombia; 4 E-Health Living Lab Faculty of Medicine Universidad de Antioquia Medellin Colombia; 5 Mental Health Department Faculty of Health Sciences Universidad Autonoma de Bucaramanga Bucarmanaga Colombia; 6 Hospital Universitario San Ignacio Bogotá Colombia

**Keywords:** Colombia, telemedicine, medical informatics, eHealth, mental health, cultural characteristics, cultural competency, ethnic groups, quality of health care, community-based participatory research, primary health care, patient participation, patient preference, patient satisfaction, consumer health information, methods, research design

## Abstract

**Background:**

Health information technologies (HITs) hold enormous promise for improving access to and providing better quality of mental health care. However, despite the spread of such technologies in high-income countries, these technologies have not yet been commonly adopted in low- and middle-income countries. People living in these parts of the world are at risk of experiencing physical, technological, and social health inequalities. A possible solution is to utilize the currently available HITs developed in other counties.

**Objective:**

Using participatory design methodologies with Colombian end users (young people, their supportive others, and health professionals), this study aimed to conduct co-design workshops to culturally adapt a Web-based Mental Health eClinic (MHeC) for young people, perform one-on-one user-testing sessions to evaluate an alpha prototype of a Spanish version of the MHeC and adapt it to the Colombian context, and inform the development of a skeletal framework and alpha prototype for a Colombian version of the MHeC (MHeC-C).

**Methods:**

This study involved the utilization of a research and development (R&D) cycle including 4 iterative phases: co-design workshops; knowledge translation; tailoring to language, culture, and place (or context); and one-on-one user-testing sessions.

**Results:**

A total of 2 co-design workshops were held with 18 users—young people (n=7) and health professionals (n=11). Moreover, 10 users participated in one-on-one user-testing sessions—young people (n=5), supportive others (n=2), and health professionals (n=3). A total of 204 source documents were collected and 605 annotations were coded. A thematic analysis resulted in 6 themes (ie, opinions about the MHeC-C, Colombian context, functionality, content, user interface, and technology platforms). Participants liked the idea of having an MHeC designed and adapted for Colombian young people, and its 5 key elements were acceptable in this context (home page and triage system, self-report assessment, dashboard of results, booking and video-visit system, and personalized well-being plan). However, to be relevant in Colombia, participants stressed the need to develop additional functionality (eg, phone network backup; chat; geolocation; and integration with electronic medical records, apps, or electronic tools) as well as an adaptation of the self-report assessment. Importantly, the latter not only included language but also culture and context.

**Conclusions:**

The application of an R&D cycle that also included processes for adaptation to Colombia (language, culture, and context) resulted in the development of an evidence-based, language-appropriate, culturally sensitive, and context-adapted HIT that is relevant, applicable, engaging, and usable in both the short and long term. The resultant R&D cycle allowed for the adaptation of an already available HIT (ie, MHeC) to the MHeC-C—a low-cost and scalable technology solution for low- and middle-income countries like Colombia, which has the potential to provide young people with accessible, available, affordable, and integrated mental health care at the right time.

## Introduction

### Background

According to the World Bank, Colombia (48 million inhabitants) [1] is defined as a middle-income country—gross domestic product (GDP) of US $314 billion [2]. However, it is one of the most unequal countries in the world (with a 2017 Gini index of 49.7) [3]. Although the country has spent 7% of its GDP on health over the past 15 years [4], only 0.08% of that spending has gone to mental health, which is the lowest of all South American countries [5]. Furthermore, although the country has a high level of nationwide health coverage (95%) [6], this is still difficult to access for ethnic minorities and Colombia’s poorest regions. This is particularly the case for rural regions where 15% of the population lives [1]. As the Colombian health system is disease centered, the continuity and the quality of care are jeopardized in these areas because of the difficulty in attracting qualified specialists [7]. In 2017, it was estimated that there were only 1003 psychiatrists in Colombia [7] and that 80% of the psychiatrists were situated within major cities, resulting in a treatment gap of more than 50% [8].

Colombia has a very young population (40% of the population is aged below 25 years and 18% of the population is aged between 15 and 24 years) [1]. According to the most recent Colombian National Mental Health Survey (NMHS; 2015), the lifetime prevalence rate of mental health disorders for adolescents aged 12 to 17 years was 7% (any disorder), and the rate of suicide attempts for this age group was 3% [8]. This survey grouped adults between 18 and 44 years; therefore, the lifetime prevalence of these disorders in young adults is not clear. In a survey conducted in Medellin in 2012, the lifetime prevalence rates for young people aged 13 to 29 years were as follows: depression, 7%; any anxiety disorder, 13%; and posttraumatic stress disorder, 4% [9]. However, there are only a few specialized child and adolescent psychiatrists in the country; most of them are located in urban areas [10,11]. Many Colombian adolescents access mental health services (outpatient and inpatient) through adult facilities, which may not be fully equipped to meet their unique needs (appropriate to the stage of illness and developmental period, youth friendly, stigma free, preventative, positive, flexible, accessible, and affordable), and this results in more alienation for this young population [12].

Given the nature of the Colombian health system and its geography, the internet holds promise in bypassing the barriers to accessing mental health care for the country’s population. This is particularly the case as Colombia has universal internet access (broadband, satellite, or microwave) [13]. A recent information and communications technology use survey revealed that 64% of households have access to the internet and that 72% of the households have at least one smartphone. Furthermore, there are more than 1500 free Wi-Fi hotspots located at major public places in the country. Colombia was one of the first countries in Latin America to propose a specific telehealth legislation (law 1419 of 2010). Its main aim is to integrate health information technology (HIT) interventions into the local health system to provide health services across all levels: promotion, prevention, diagnostic, treatment, rehabilitation, and health education [14].

Telemedicine in Colombia has been successfully operating since 1998 [15]; presently, the country has more than 2500 registered telemedicine service centers, which are located in the major cities and towns [16]. The number of these centers is constantly growing as some of the most important academic institutions and hospitals (public and private) are committed to delivering clinical assessments (including most of the medical specialties) to rural areas and marginalized populations [14,16,17]. The delivery of asynchronous telemedicine, which involves delivering text messages to end users (more commonly containing questions) and to experts (teleconsultation), has been postulated as an effective method for providing reliable health information and open dialogue about sensitive topics such as sexuality, drug use, or health concerns in the country [18-21]. Although HITs in Colombia seem to have a positive impact, most of the interventions still require rigorous evaluation [17].

However, although telemedicine has seen success in Colombia, there are a number of barriers to its further and more integrated implementation into Colombian health care. There is still a certain degree of skepticism in the general population toward delivering health care in this way, and health professionals still have limited knowledge on how to work effectively with technology [14,22]. Notwithstanding progress in the legislation, current law still restricts the use of telemedicine in rural populations (thus limiting its use in medium and small towns) and limits the use of telemedicine as a tool to only when face-to-face contact is not available [14,22]. Other legal limitations include the need for health professionals to be on both sides of the assessment (institution of remission and institution of reference), meaning that an individual cannot directly connect with local or international health professionals, and there are some concerns related to security, privacy, data sharing, and data integrity [14,22]. Innovative uses of HITs, such as mobile health, and ubiquitous health, are still unregulated.

These barriers contribute to lack of uptake, engagement, and adherence, as well as high dropout rates. These phenomena can be explained by Eysenbach’s attrition law [23], which postulates that a substantial proportion of end users lose interest or experience some difficulties while using the technological intervention and thus stop using it. This might be because of the perception that the intervention is not creating any benefit, that it is responding to an overly specific need, or that it has usability problems [23]. Although academia-led HITs have the strength of incorporating evidence-based and best clinical practices into their design, it is common to sacrifice the intervention’s usability over content because of limited funding [11,24]. For researchers, it is hard to compete with commercial products that provide highly intuitive and engaging experiences in their products, despite having unknown evidence-based or clinical value [11,24].

To ensure that end users of HITs can derive maximum value from such interventions, it is critically important to involve them in their design and development and to strike a balance between best clinical practice and user experience (including usability). Participatory design (PD) methodologies represent one such solution [25-27]. The process involves engaging end users and other stakeholders at all stages (from conception to completion) of the design, development, and testing of these technologies [26,28,29]. Through several iterative phases, the prototype is co-designed, codeveloped, and refined until it has value to the end users; meets their needs; and is appealing, engaging, acceptable, and usable [26,30,31]. As end users share equal responsibility with the researchers for the outcomes, the rationale behind the use of PD methodologies could result in better products that are more functional in real-life settings, thereby closing the translational research gap [26]. In recent years, it has become more common to see the use of these methodologies in the development of mental health interventions in English-speaking countries [26-28,30,32]. However, to our knowledge, these methodologies have not yet been used in Colombia or any other Latin American country in this field.

The University of Sydney’s Brain and Mind Centre (BMC) is a leader in the development of evidence-based electronic health technologies [11,26,33-38]. Through a partnership with the Young and Well Cooperative Research Centre (2014-2016), the prototypic version of the Mental Health eClinic (MHeC) [26,36] was designed and developed. This Web-based tool aimed to deliver best-practice clinical services to people experiencing mental health problems, making clinical care accessible, affordable, and available to young people whenever and wherever they need it most. The original MHeC was then co-designed and culturally adapted, developed, and user tested (2015-2017) with Spanish-speaking young people currently living in Australia, resulting in the Spanish version of the Mental Health eClinic (MHeC-S) [31].

The original MHeC comprised 5 key elements: a home page with a visible triage system for those requiring urgent help, a comprehensive Web-based physical and mental health self-report assessment, a detailed dashboard of results (with colored icons and traffic light representations of results), a booking and videoconferencing system to enable video visits, and the generation of a personalized well-being plan that includes links to evidence-based apps and e-tools recommended by health professionals and suggested by young people [26]. These elements were well accepted by Spanish-speaking young people living in Australia [31]. Considering the potential of the MHeC-S to be configured and adapted for use in Spanish-speaking countries and in other multicultural countries with Spanish-speaking migrant populations, as well as Colombia’s health and internet characteristics described above, we envisioned that a Colombian version of the MHeC (MHeC-C) could greatly benefit young Colombians who are actively seeking help.

### Aims

Using a modified version of our already established research and development (R&D) cycle [26,31] with Colombian end users (young people aged 16 to 30 years, supportive others, and health professionals) as a framework, this study aimed to (1) conduct co-design workshops with end users to culturally adapt the MHeC for young people in Colombia, (2) perform one-on-one user-testing sessions with end users to evaluate the alpha prototype of the MHeC-S and how to adapt it to the Colombian context, and (3) inform the development of the skeletal framework and alpha prototype of the MHeC-C.

## Methods

### Participants

Participants included community-based young people aged 16 to 30 years, health professionals, and supportive others with regular access to a mobile phone (iPhone or Android) and the internet. The recruitment strategy included the identification of potential participants through the reference groups and youth reference groups of our Colombian partner institutions (Pontificia Universidad Javeriana, Universidad de Antioquia, and Universidad Autónoma de Bucaramanga), posters and postcard advertisements displayed in common areas where the reference groups meet, Facebook advertisements, and a study-specific Facebook page.

The University of Sydney’s Human Research Ethics Committee approved this study (protocol number 2014/689 for the co-design workshops and protocol number 2016/487 for the user-testing sessions); however, as requested by the Human Research Ethics Committee, local (Colombian) approvals were also obtained to ensure that the study complied with all the local regulations on research with humans. Participants were provided with relevant information about the study (participant information statement) before consenting and participating in the study. Young people were provided gift vouchers to thank them for their time and expertise when they attended co-design workshops and user-testing sessions.

### Research and Development Cycle

**Figure 1:**
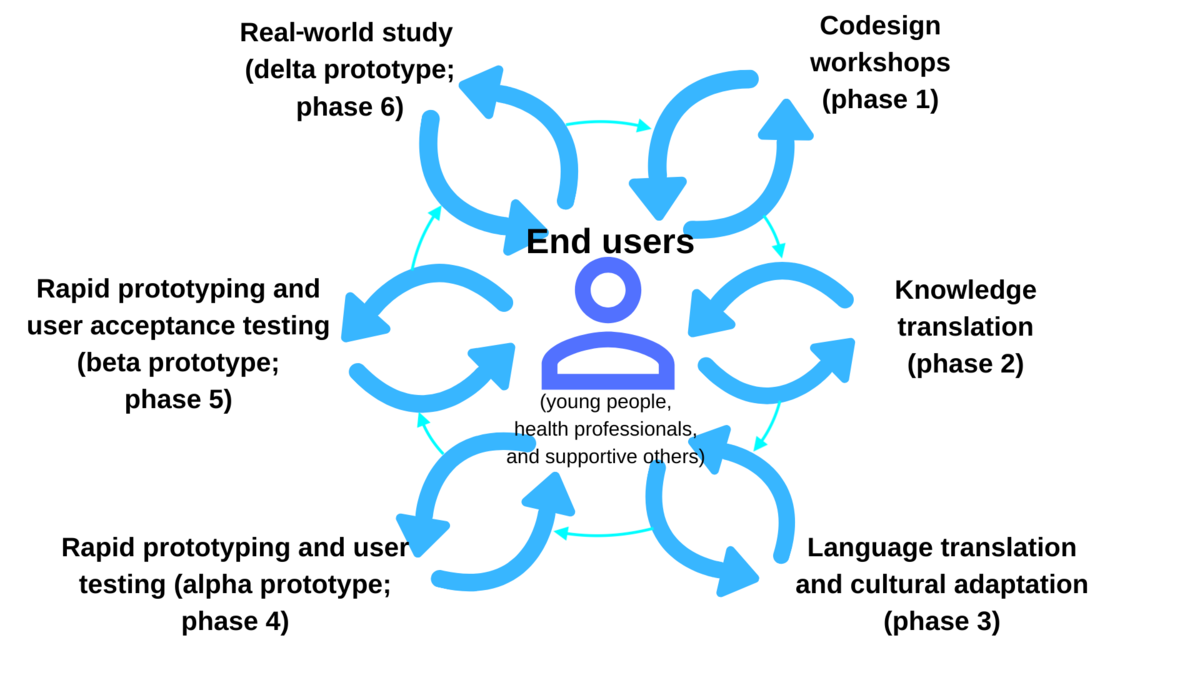
Previously established research and development cycle of the Spanish version of the Mental Health eClinic.

**Figure 2:**
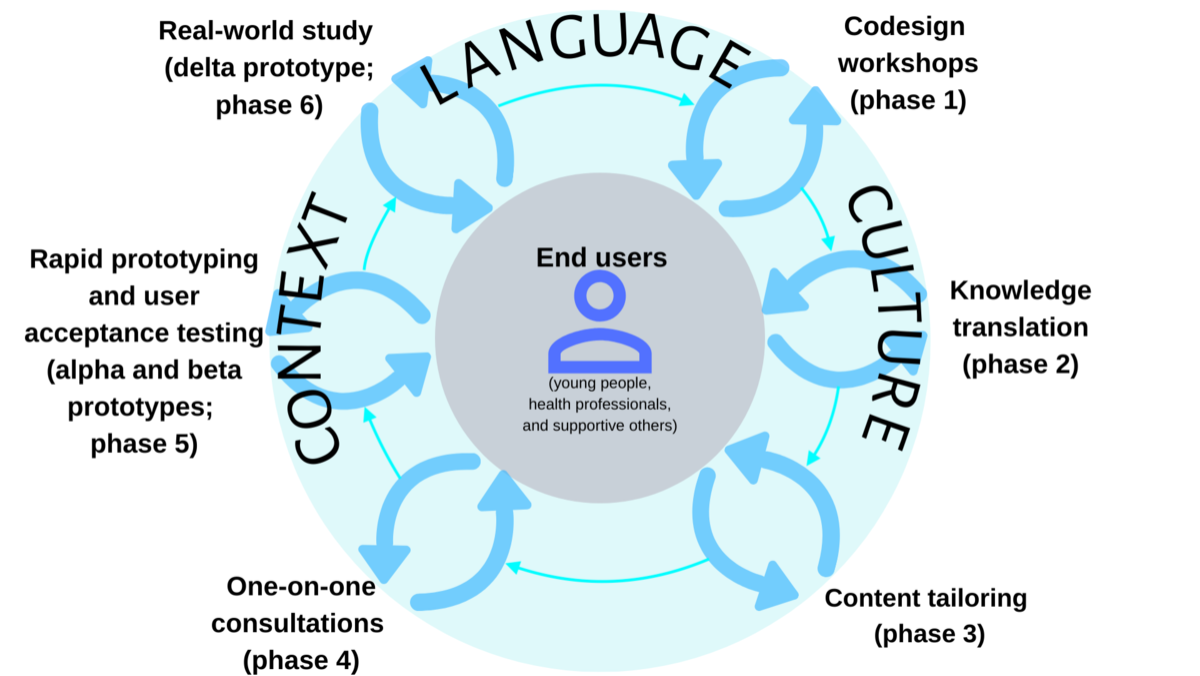
A language-appropriate, culturally sensitive, and contextually adapted framework as an intrinsic part of the research and development cycle.

The PD methodologies used in this study were based on the guidelines provided by the Young and Well Cooperative Research Centre [39] and were similar to the ones applied in our previous research [26,30,31]. The R&D cycle implemented in this study has been demonstrated to be an efficient method to obtain the most information from end users by engaging them in different activities. For this exploratory study, we conducted a modified version of our previously established R&D cycle (Figures 1 and 2) [26,31]. This study comprised 4 concurrently running phases: co-design workshops (phase 1), knowledge translation (phase 2), content tailoring (phase 3), and one-on-one user-testing sessions (phase 4). Considering that language and culture are the key aspects in the process of adaptation, we decided to incorporate language and culture as part of the framework the R&D cycle is based on. With that in mind, phase 3 (language translation and cultural adaptation) [31] of our previous MHeC-S’s R&D cycle moved to be the cornerstone of the cycle used in this study, and phase 3 in this study only refers to the content tailoring process. Phases 5—rapid prototyping and user testing (alpha, a preliminary version that can be interacted with for user-testing purposes, and beta, a more refined version of the prototype that is much closer to the final product, prototypes)—and 6—real-world study, with a delta prototype that can be used directly by end users for feasibility testing—would be the subject of future research.

### Phase 1: Co-Design Workshops

We held 2 co-design workshops, one with young people and the other one with health professionals. The workshops were conducted in Bogota, Colombia, in 2015. The aim of these workshops was to identify how best to co-design the MHeC-C’s alpha prototype and, broadly, how to adapt the MHeC to a Colombian setting and population. The half-day (4 hour) workshops comprised 3 stages: discovery, evaluation, and prototyping. At the end of each workshop, the information was analyzed and synthesized by a knowledge translation team (comprising 2 interns at The University of Sydney’s BMC) for design testing in subsequent workshops. Digital technology was not used in any stage of the workshops.

#### Discovery

Workshop moderators facilitated participant discussion in relation to the following topics: defining the advantages and disadvantages of having an MHeC-C, defining the barriers of having an MHeC-C, and establishing how a prototype like this should look and function to meet the young persons’ needs in the Colombian context. Handwritten notes were taken during the entire workshop.

#### Evaluation

Participants were then presented with screenshots of existing mental health websites and wireframes or mock-ups of the early versions of the MHeC and the MHeC-S for their critical evaluation. These items contained a variety of features of interest, such as the 5 key elements of the MHeC and other relevant apps and e-tools related to mental health or well-being. Marker pens were provided for participants to annotate their observations.

#### Prototyping

Finally, participants were asked to hand-draw their ideas, specifications, and requirements for an MHeC-C. Sketchbooks and marker pens were provided for this activity.

### Phase 2: Knowledge Translation Process

The knowledge translation process comprised analyzing the visual artifacts (mock-ups and end-user sketches) produced in the design-testing and sketching stages and tallying requested MHeC-C features from the notes taken in phase 1 (co-design workshops). Observations that were repeated 3 or more times were considered for inclusion in phase 4 or in the development of wireframes. The discrepancies that arose during this process were discussed between the knowledge translation team and 2 mental health researchers and Colombian psychiatrists (LOP and ANM) until reaching consensus in the second session.

### Phase 3: Content Tailoring

LOP and ANM reviewed the general content of the MHeC-S alpha prototype to detect language subtleties. A literature review of published (identified via PubMed, Google Scholar, Scientific Electronic Library Online, and Latin American & Caribbean Health Sciences Literature) and gray literature (identified via Google Advanced search) was undertaken by LOP to identify relevant measures for this population, as well as those instruments already translated, validated, and used in Colombia. Recognizing that some questionnaires might have several versions, the following process was established to select instruments: (1) selection of official and published translations and (2) selection of published Colombian versions of the official translations. When more than 1 version or source was available, the 2 Colombian psychiatrists (LOP and ANM) selected the most appropriate version or source to be included through discussion and consensus. If the questionnaires were not publicly available or there were no self-report versions for the topics to be assessed, expert recommendation (discussion and consensus among 3 Colombian psychiatrists LOP, ANM, and AC) was utilized.

### Phase 4: Remote One-on-One User-Testing Sessions

Phase 4 involved in-depth one-on-one user-testing sessions with new end users (young people, health professionals, and supportive others). The sessions were held remotely using GoToMeeting and its shared screen capacity (GoToMeeting by LogMeIn, Boston, Massachusetts, United States, is a screen sharing software that allows users to display the entire screen, multiple monitors, or specific apps at any time) [40], using laptops, tablets, and mobile phones. In each 90-min one-on-one user-testing session, a researcher guided an end user into the already available alpha prototype of the MHeC-S. Using a think-aloud protocol [41], participants provided their observations as they were shown the navigation through the prototype. These sessions also explored the utility and the end users’ inclination to use an MHeC in Colombia, overall comments, and the process of naming the prototype. Handwritten notes were taken during all sessions.

### Data Analysis

All source documents (phase 1, co-design workshop notes and artifacts, and phase 4, user-testing notes) were uploaded to NVivo 11 for Mac (QSR International) and analyzed using thematic analysis techniques [42,43]. Importantly, source documents were analyzed at the end of each phase to explore preliminary findings and inform subsequent phases. The thematic analysis framework involved both inductive and deductive coding, with the deductive codes being 5 previously identified themes [31]: help-seeking barriers, technology platform, functionality, content, and user interface [26]. A total of 2 Colombian psychiatrists (LOP and ANM) coded the material, and 1 researcher analyzed the information (LOP). Data collection and qualitative analysis were conducted in Spanish by LOP and ANM. To facilitate the reporting of results, translated quotes from the source documents are included below, and Multimedia Appendix 1 lists the original quotes in Spanish.

## Results

### Co-Design Workshops and User-Testing Sessions

In June 2015, we conducted 1 half-day co-design workshop with young people in Colombia and 1 half-day co-design workshop with Colombian health professionals. In total, we conducted 2 knowledge translation sessions: one after the co-design workshops (phase 1) and the other at the end of the one-on-one user-testing sessions (phase 4). We conducted 10 remote one-on-one user-testing sessions in August 2017. The language and cultural adaptation process started in June 2015 and finished in November 2017.

### Participant Characteristics

A total of 7 young people participated in the co-design workshops; 5 were female, and their ages ranged from 18 to 22 years (median age 19.5 years). A total of 11 health professionals participated in the workshops; 5 were female, and their ages ranged from 20 to 29 years (median age 27 years). Of the health professionals, 2 were medical students and the rest were psychiatry registrars (Table 1).

A total of 10 participants participated in the one-on-one user-testing sessions: 5 young people with ages ranging from 17 to 24 years (median age 22 years), 3 health professionals with ages ranging from 29 to 36 years (median age 29 years; all of them were psychiatrists), and 2 supportive others with ages ranging from 19 to 24 years (median age 21.5 years). Of these participants, 7 were female (Table 1).

**Table 1:** Participants’ characteristics.

Characteristics		Co-design workshops with young people (n=7)	Co-design workshops with health professionals (n=11)	One-on-one user-testing sessions (n=10)
**Demographics**				
	Female, n (%)	5 (71)	5 (45)	7 (70)
	Age (years), median (IQR)^a^	20 (2)	27 (1.5)	23 (6.5)
**Education**				
	Secondary, n (%)	7 (100)	2 (18)	6 (60)
	Tertiary, n (%)	0 (0)	9 (82)	4 (40)

^a^IQR: interquartile range.

### Coding Framework

During the co-design workshops, a total of 194 source documents were developed and analyzed (2 sets of workshop notes and 192 artifacts produced by participants). A total of 312 annotations were coded: 106 annotations in the content theme, 151 annotations in the functionality theme, and 47 annotations in the user interface theme. Moreover, 2 new themes emerged in this phase: opinions about the MHeC-C (4 annotations) and Colombian context considerations (4 annotations). There were no annotations in the help-seeking or the technology platform themes in this stage.

During the one-on-one user-testing sessions, 10 sets of notes were generated. A total of 293 annotations were coded: 132 annotations in the functionality theme, 58 annotations in the user interface theme, 42 annotations in the content theme, 23 annotations in the opinions about the MHeC-C theme, 20 annotations in the Colombian context considerations theme, and 18 annotations in the technology platform theme. There were no annotations in the help-seeking theme; consequently, it was removed from the coding framework analysis.

For the purposes of this paper, we report the data aggregated from the co-design workshops and the one-on-one user-testing sessions, specifying in which session the information was collected where relevant.

#### Opinions About the Colombian Version of the Mental Health Electronic Clinic

All participants (28/28) liked the idea of having an MHeC specially designed for and adapted to a Colombian context. As possible advantages, they suggested it would reduce costs even if the initial investment would be considerable, and in the long run, individuals would save time and money and the need for physical infrastructure would be less. All young people (12/12), all health professionals (14/14), and supportive others (2/2) agreed that a prototype like this would expand access to health professionals (especially in rural areas), facilitate monitoring, and reduce loss to follow-up. This would ultimately increase satisfaction, convenience, and engagement with the health system, as individuals would have more flexibility with their time and no location barriers. In addition, all health professionals (14/14) felt the prototype would improve the health service network, as it would provide specialized assessments, regardless of the individuals’ location, and support for rural professionals. Integrating the MHeC-C with electronic medical records, laboratory results, and pharmacological records would increase treatment adherence and provide more objective information that would translate to better monitoring and health outcomes. Some health professionals from the co-design workshops (7/11) also believed that this prototype could be safer in cases of assessing individuals with violent behaviors, whereas the rest (6/11) believed that they would feel safer if the MHeC-C was part of the already established health network.

However, regarding disadvantages and barriers, all participants (28/28) mentioned that in some places, the internet connection is not reliable, so the prototype needs to be backed up with a phone network. Among barriers of using an MHeC-C, all young people (12/12) mentioned difficulties while accessing the internet, as most young people do not pay for mobile data and therefore require internet access at their homes and schools, or they require free Wi-Fi networks. All health professionals (14/14) recognized that the MHeC-C could have limited utility in acute cases or in cases where performing physical (neurological) assessments would be required.

#### Colombian Context Considerations

Overall, health professionals (14/14) believed that the MHeC-C should be led by a partnership between a university and a health service provider and have strong networks with the community and other relevant organizations. A partnership with local governments and stakeholders would be necessary but especially relevant in rural settings to increase trust and, as such, increase the acceptability of the prototype. For people to be able to use the MHeC-C, it needs to be recommended by clinicians, health services, and school and university well-being centers, which should be complemented with publicity and media coverage (eg, radio, television, social networks, magazines, and newspapers). As most young people are not economically independent, it would be important for the MHeC-C to be embedded in the public health care system.

In relation to the branding and name of the MHeC-C, young participants (12/12) considered that the combination of terms *mental health* and *clinic* would be less appealing for them, as they might feel that the MHeC-C only deals with severe cases and might not be appropriate for them and that it would consequently be more stigmatizing.

#### Functionality

As defined by Valdez et al in their culturally informed design framework [44], functionality indicates the actions that can be performed in the prototype. All participants (28/28) agreed that the 5 key elements of the MHeC-C were acceptable in this context. In general, participants agreed that the MHeC-C should be compliant with international cybersecurity standards to ensure privacy and data protection.

#### Element 1: Home Page and Triage System

All participants (28/28) agreed that to gain trust and increase credibility, the MHeC-C’s webpage domain should be *.com*, *.co*, or *.org.* Alternatively, the MHeC-C could be imbedded in universities’ official websites, as they believe universities should have a lead role in the development and maintenance of this kind of prototype. The logos of the principal institutions as well as partner organizations should be displayed at this stage. Participants also agreed with providing a small description of the MHeC-C, delivered with images, videos, and testimonials from young people and health professionals. Both young people and health professionals agreed that the initial home page could be the same for both groups; however, after registration and log-in processes, the prototype would change to address both user types’ different needs.

All participants liked the triage functionality and recognized the importance of promptly referring someone to the emergency help services. In the same line, the *Need Help Now* button was identified as an important resource for people in crisis who were unaware of the emergency lines. Participants highlighted the importance of this button to be associated with a geolocation system, as in Colombia, emergency (psychological) numbers change according to their location. A health professional explained the following:

...the general emergency line is the same 123, but the psychological emergency line changes, for example in Bogota it is 106 and in Cartagena it is 125...

Health professional, quote A

**Figure 3:**
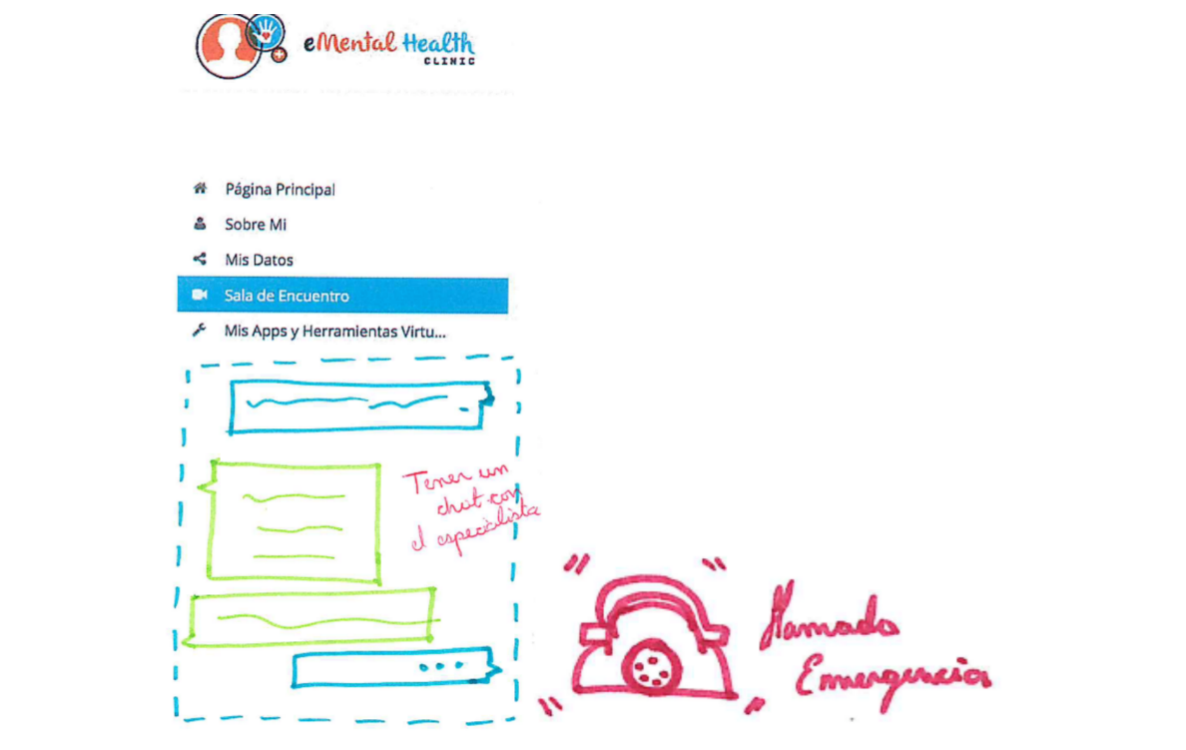
Hand-drawn sketch by end users during a participatory design workshop representing the chat functionality and the phone support service.

As Web-based services are scarce in Colombia, it was proposed to have a 24/7, moderated Web-based chat that would provide support and counseling to individuals seeking help. For young people, this functionality would be situated under the *Need Help Now* button. Health professionals believed that a functionality like this would also be useful for them to provide guidance and supervision to other less experienced health professionals (eg, general practitioners in their social compulsory service) or to those located in rural areas. The chat functionality for health professionals would work only for health services and professionals attached to the MHeC-C. In case the internet connection is intermittent or lost, the chat functionality should also have a phone support service that would be enabled to continue with the conversation (Figure 3).

**Figure 4:**
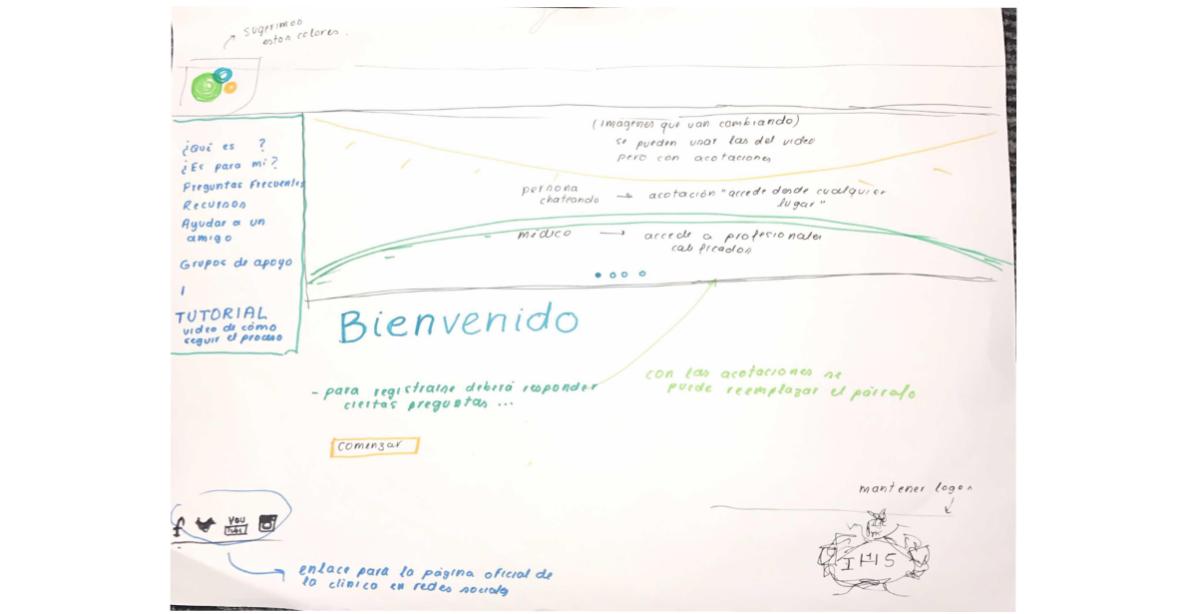
Hand-drawn sketch by end users during a participatory workshop representing the home page.

**Figure 5:**
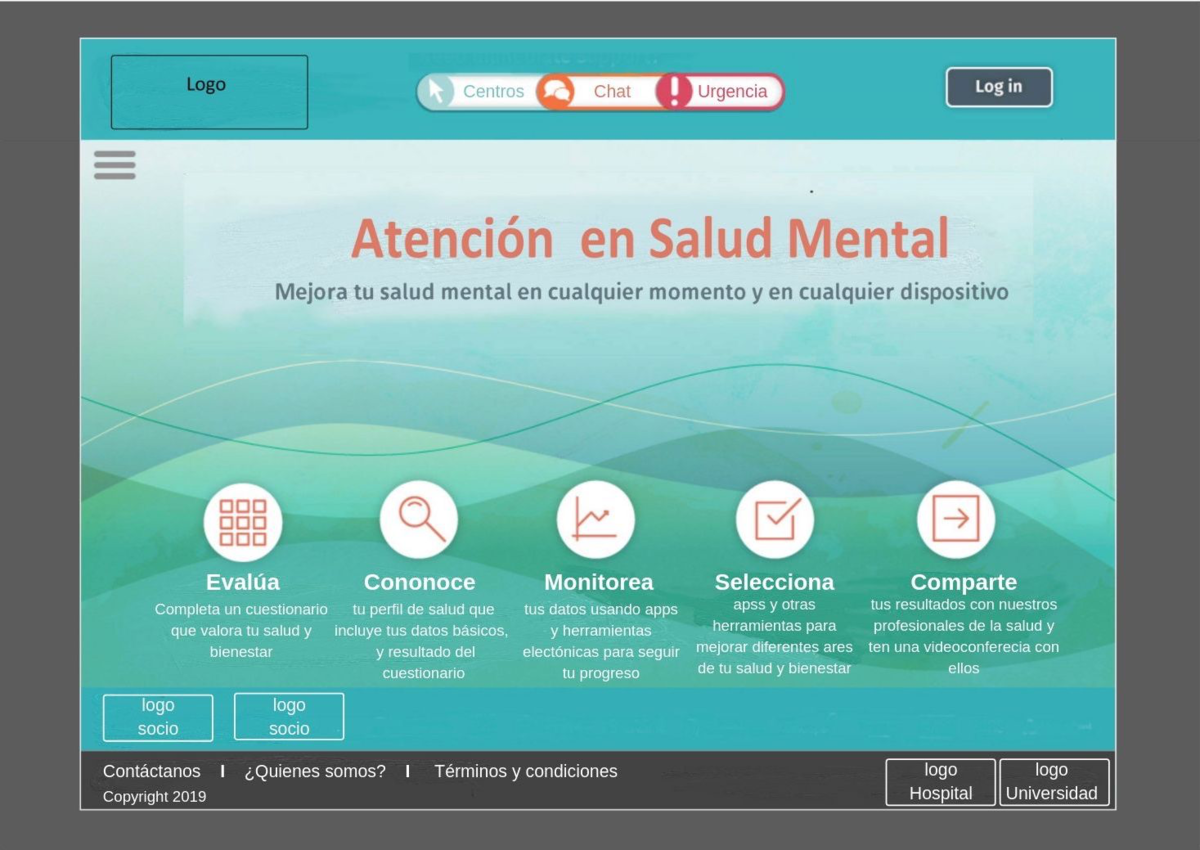
Screenshot of the skeletal framework of the home page of the Colombian version of the Mental Health eClinic.

Participants acknowledged the difficulty of having health professionals available at all times to chat; therefore, they proposed that the chat should work only during extended hours (from 6 am to 12 am), and in off-time hours, they should have the option to leave a question to be answered later. At the same time, young people recognized the importance of having carefully moderated blogs, forums, or group chats with a selection of helpful topics to find support and learn from other people’s experiences. Figures 4 and 5 represent the proposed home page for future developments.

#### Element 2: Web-Based Physical and Mental Health Self-Report Assessment

All participants agreed with the need to assess young people’s physical and mental health. The already established features of this element were accepted among the end users: modular display of question sets, capacity of pausing and resuming later, and rule-based decision algorithms that enable a personalized assessment of the young person. Again, participants mentioned the possibility of using geolocation to automatically collect data about the participants and personalize the assessment. A health professional explained the following:

...it would be very useful to geolocate the person, this means the prototype would be able to know where they are so they don’t have to waste time filling their addresses. Also, as Colombia is so diverse, we know that the regions have different needs so the questions could be specific to those needs. For example, in regions affected with violence, assessing this topic in-depth would be crucial. Another example would be assessing thoroughly the social determinants of health if the person lives in a poor area or is identified with a low socioeconomic status...

Health professional, quote B

The types of questions in the prototype (Likert-type scale questions and 2-way close-ended questions) were also acceptable to participants. However, health professionals (11/14) suggested adding visual responses, such as the pain visual analog scale [45], and including 1 open-text question with the aim of assessing the individual’s reason for accessing the MHeC-C over traditional face-to-face services.

#### Element 3: Dashboard of Results and Progress Report

There was a discrepancy in the end users’ opinion on the immediate display of the dashboard of results after completion of the Web-based self-report assessment. All young people (12/12) and some health professionals (6/14) agreed that the prototype should display the results immediately. Other health professionals (8/14) were concerned with the pertinence of the results, as a young person could potentially experience some distress while viewing their results, especially for those living in rural areas. As a potential solution to this, participants suggested giving individuals the option to pick if they want to see their results immediately or wait to review their results with a health professional.

Participants agreed with the traffic light representations and colored icons. Simple bar and line graphs were preferred to represent progress and track data over time. Health professionals considered that the dashboard of results was useful to inform their practice, making the assessments more efficient and specific as well as enabling them to deliver the interventions earlier and monitor the individual’s progress over time. In addition, health professionals believed that the results of the assessment and the dashboard were useful research tools. In relation to the dashboard’s language, lay terms were preferred over medical terminology. The option of displaying a simple explanation of the term (only when medical terms are needed) when participants click on the word or hover over it was widely accepted among the participants.

#### Element 4: Booking System and Video Visit

Before booking a video visit, participants wanted to view the profiles of the health professionals attached to the MHeC-C so that they could choose the professional they want to see. A young person explained the following:

...I would like to know more who I’m going to see, so I can decide if I see a man or a woman or see what are their areas of expertise...

Young person, quote C

In addition, it was proposed to have calendar functionality so that young people could book appointments according to the health professionals’ availability. This functionality should also reflect other relevant calendars, such as the health professional’s calendar, and the administrative staff so they can use it for other purposes such as billing.

Health professionals considered the video visit to be a useful tool for providing supervision, training, and consultation to colleagues located in rural areas. A health professional explained the following:

...doctors in their social compulsory service (located in rural areas) might need support from specialists, it would be very useful to use the video visit system to help them assessing difficult cases or to provide supervision...

Health professional, quote D

In addition, as some health services still have paper-based medical records, having an electronic medical record attached to the MHeC-C would be ideal so that all the individuals’ information could be stored in the same place.

Given that health professionals would have detailed and accurate self-report information before the video visit (dashboard of results), all participants agreed that around 20 mins would be enough time to assess a young person and provide recommendations. Health professionals would also like the possibility to extend video-visit time with complex cases. Should a video-visit appointment run late, health professionals also suggested that the MHeC-C should send a notification to people waiting for subsequent appointments.

#### Element 5: Personalized Well-Being Plan Includes Links to Evidence-Based, Young Person–Suggested and Health Professional–Recommended Apps and E-Tools

Participants accepted the activation of a personalized well-being plan and recommendations according to their results. Young people and health professionals believed that these recommendations could be delivered as apps, videos, or printable material. Health professionals suggested the MHeC-C could be connected to the website *mental punto de apoyo* [46,47], as this informational website has a wide variety of information for individuals, supportive others, and health professionals; as well as, psychoeducational and training material; and community blogs.

The issue about shortage of Spanish-language apps and e-tools was also raised. Health professionals believed that developing such apps to track variables such as mood, sleep, physical activity, and nutrition as well as interventional apps that contain cognitive behavioral therapy strategies and mindfulness would be necessary. In general, participants believed these apps and e-tools need to be in Spanish, as the chances of using an English-based app are minimal. The need to create videos with general information, as well as relaxation and breathing exercises, was also mentioned.

### Content

#### General Content

Content refers to the message that is transmitted [44]. Participants from the one-on-one user-testing sessions had the opportunity to explore the alpha prototype of the MHeC-S. These participants (10/10) found that some pieces of general content already available were relevant for them but needed minor tweaks to fit the context, such as general information about the MHeC-S, breathing exercises, frequently asked questions, and how to help a friend. Other content including health services information, terms and conditions, and information about partner organizations needed major changes to be relevant in Colombia. Again, the scarcity of Spanish-language apps and e-tools was highlighted, as they are the cornerstone of the personalized well-being plan.

#### Cultural Adaptation of the Self-Report Assessment

The original Spanish-language self-report assessment included 20 modules (Table 2) with smart skips built in so that it was tailored to each individual and took the minimum possible amount of time to complete (approximately 45 min) [31].

Of the 20 modules, 19 modules were considered relevant by the participants and 1 module (cultural adaptation and adjustment disorder) was considered unnecessary. Health professionals (3/3) and supportive others (2/2) from the one-on-one user-testing sessions suggested including further topics to be assessed. As *family* is very important in the Colombian culture, it was suggested to assess family structure and support network. Religion and spirituality were also considered as important factors to be assessed, as they might influence an individual’s mental health, act as support, or define some treatments. Owing to the country’s characteristics, it was also considered necessary to evaluate social risk by screening economic stability, neighborhood and physical environment, food security, and access to the health care system [83]. As Colombia has been severely affected by violence, participants also suggested to evaluate violence exposure, trauma, and resilience.

**Table 2:** Self-report assessments in the Spanish version of the Mental Health eClinic and the Colombian version of the Mental Health eClinic.

Module and questionnaires	Self-report assessments		
	MHeC-S^a^ and MHeC-C^b^	MHeC-S only	MHeC-C only
Main reason for visiting	Short open-text question	—^c^	—
General demographics		Items adapted to Spanish from the Second Australian Young and Well National Survey [48] and the 2-step method to measure transgender identity [49]	Items adapted to Spanish from the Second Australian Young and Well National Survey [48] and the 2-step method to measure transgender identity [49]. Religion, spirituality, socioeconomical status, food insecurity, sanitation, access to drinking water, electricity, housing, assets, and health care selected items from the NMHS^d^ [50]
Social and occupational function	World Health Organization Disability Assessment Schedule 2.0 [51] and an adapted version of the self-report version of the Social and Occupational Functioning Assessment Scale [52]	—	—
Psychological distress	10-item Kessler Psychological Distress Scale [53]	—	—
Depressed mood	QIDS-SR-16^e^ [54,55]	—	—
Anxiety	Generalized Anxiety Disorder Assessment-7 [56]	—	—
Mania-like experiences	Items derived from the Altman Self-Rating Mania Scale [57]	—	—
Psychosis-like experiences	Items derived from the Community Assessment of Psychic Experiences-Positive Symptoms Scale [58,59]	—	—
Traumatic experiences		Primary Care PTSD^f^ Screen [60] and the PTSD Checklist-Civilian Version [61]	Attitudes and experiences to violence (domestic violence, organized crime, displacement, and armed conflict) from the NMHS. Selected items from the Adverse Childhood Experiences [62]. Primary Care PTSD Screen [60] and the PTSD Checklist-Civilian Version [61]
Self-harm behaviors and suicidal ideation	Suicide Behaviors Questionnaire-Revised [63]	—	—
Tobacco, alcohol, and substance use	Items adapted from Alcohol Use Disorders Identification Test [64]; Alcohol, Smoking, and Substance Involvement Screening Test [65]; and Cutting down, Annoyance by criticism, Guilty feeling, and Eye-openers questionnaire [66]. Items adapted to Spanish from the Drinking Motives Questionnaire [67], Fagerström Test for Nicotine Dependence [68], and selected items adapted to Spanish from the National Drug Strategy Household Survey [69]	—	—
Physical activity	International Physical Activity Questionnaire [70,71]	—	—
Sleep behaviors	Sleep-related items from the QIDS-SR-16		
General mental health conditions	Spanish version of the World Mental Health Composite International Diagnostic Interview used in the National Comorbidity Survey Replication Adolescent Supplement [72,73]	—	—
Overall heath and somatic distress	Items adapted to Spanish from the Somatic and Psychological Health Report [74], self-perceived health status, and general body measurements	—	—
Medical, mental health, and family history	Multiple-choice questions	—	—
Cognitive concerns and empathy	Items derived from the Subjective Scale to Investigate Cognition in Schizophrenia [75], adapted to Spanish, and the empathy quotient [76]	—	—
Eating behaviors and body image	Items derived from the Eating Disorder Examination [77], adapted to Spanish	—	—
Social connectedness and support (and family structure for Columbian version)	—	Items derived from the Perceived Social Support/Conflict Measure [78], plus 5 items measuring relationships with peers [79], adapted to Spanish	Items derived from the Perceived Social Support/Conflict Measure [78], plus 5 items measuring relationships with peers [79], adapted to Spanish, and family APGAR [50,80]
Cultural adaptation and adjustment disorder	—	The Brief Sociocultural Adaptation Scale, the Brief Psychological Adaptation Scale, the Brief Perceived Cultural Distance Scale, and the Brief Acculturation Orientation Scale [81]	—
Resilience	—	—	Connor-Davidson Resilience Scale (CD-RISC 10) [82]
Cultural adaptation and adjustment disorder (optional, consider in the case of migrant populations)	—	—	The Brief Sociocultural Adaptation Scale, the Brief Psychological Adaptation Scale, the Brief Perceived Cultural Distance Scale, and the Brief Acculturation Orientation Scale [81]

^a^MHeC-S: Spanish version of the Mental Health eClinic.

^b^MHeC-C: Colombian version of the Mental Health eClinic.

^c^Not applicable.

^d^NMHS: National Mental Health Survey.

^e^QIDS-SR-16: Quick Inventory of Depressive Symptomatology-16.

^f^PTSD: posttraumatic stress disorder.

The cultural adaptation of the self-report assessment started in November 2016, with the literature review. We found 6 questionnaires that could be integrated to the MHeC-C to address the already mentioned needs. To assess family structure and support network, we selected the family APGAR, which has been widely used in Colombia [50,80]. To assess social risk, we selected items assessing socioeconomical status, food insecurity, sanitation, access to drinking water, electricity, housing, assets, and health care from the NMHS [50]. Items regarding attitudes toward and experiences with violence (domestic violence, organized crime, displacement, and armed conflict) from the NMHS were also included. Select items from the Adverse Childhood Experiences questionnaire were selected to enrich the trauma component [62]. In relation to resilience, we found 3 scales validated in the Colombian context—Adolescent Resilience Scale [84], Child and Youth Resilience Measure 12-item [85], and Connor-Davidson Resilience Scale (CD-RISC 10) [82]. All these scales assess the internal sources of resilience [86]; however, the last 2 assess external resources as well. We selected the CD-RISC 10 because of its length and because it has been widely used in the country. Religion and spirituality were also assessed with selected items from the NMHS. Table 2 represents the proposed self-report assessment for the MHeC-C.

#### User Interface

User interface refers to the visual presentation of content and functionality [44]. When shown the home page, participants agreed that the website should not only look professional but also be appealing and engaging for a young person. Horizontal menus were preferred over vertical menus in a laptop interface, but hamburger and vertical menus were the preference in tablets or mobiles. Young people (12/12) preferred to have less text and more visual content. Health professionals (14/14) and supportive others (2/2) also recognized the importance of visual content, as they believed that young people tend to read just the minimum amount of text and that information could be lost. Participants preferred to have on the home page pictures of young people interacting with the MHeC-C, with a light background or calming landscape.

**Figure 6:**
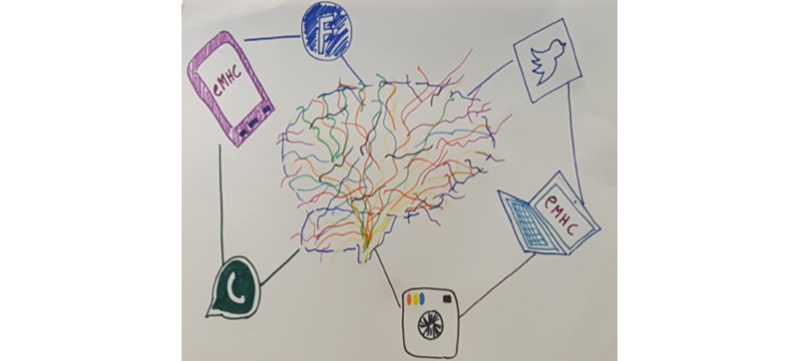
Hand-drawn sketch by end users during a participatory workshop representing the Colombian version of the Mental Health eClinic’s logo.

The color palette suggested in the co-design workshops was blue-green complemented with yellow-orange. However, participants from the one-on-one user-testing sessions liked the orange color. The MHeC-S logo was rejected by participants in the one-on-one user-testing sessions, as they did not find any representation of mental health on it and did not find the color appealing. Most participants (24/28) suggested a logo depicting a brain or a head (Figure 6):

...It reminds me of orange uniforms of the Colombian Civil Defense...

Young person, quote E

...I might be wrong but the logo needed to include a brain or a head or something like that...

Health professional, quote F

Participants felt that the *Need Help Now* button needed to draw individuals’ attention, and they suggested making this button bigger or brighter and perhaps adding an icon that represented help, such as a ringing phone, a Christian cross, or an SOS acronym. Participants also felt that *Need Help Now* should provide chat functionality and information about local emergency phone lines.

In relation to the interface’s language (regarding formal and informal pronoun usage), all end-user groups agreed that the preference to use a particular pronoun was not an issue; however, they highlighted the importance of using the pronouns consistently. A health professional explained the following:

...the country is so diverse that there are regions that use formal pronouns and others informal pronouns, the most important thing is to use it consistently...

health professional, quote G

As a possible solution to reconcile this discrepancy, it was proposed that the prototype should use the colloquial or familiar form of the second-person singular pronoun (in Spanish: *tú*), as it was targeting young people.

#### Technology Platform

Technology platform refers to the different types of hardware [44] the prototype should work on. Unanimously, participants agreed that mobile phones were the most important device to increase the reach of young people. However, health professionals also suggested that it should work on desktops, laptops, and tablets, which are their preferred devices in the workplace.

## Discussion

### Principal Findings

This exploratory study used a modified version of our previously established R&D cycle to co-design and culturally adapt a prototypic Spanish-language version of a Web-based MHeC-S into a Colombian version for young people in Colombia (MHeC-C). A thematic analysis resulted in adequate acceptability of the functionality of the 5 key elements of the prototype (a home page and triage system; a comprehensive Web-based physical and mental health self-report assessment; a dashboard of results and progress report; a booking and videoconferencing system to enable video visits; and the generation of a personalized well-being plan that includes links to evidence-based, young person–suggested, and health professional–recommended apps and e-tools). However, for these elements to be relevant in Colombia, participants stress the need to develop additional functionalities, such as backing up the system with a phone network, a chat system, a geolocation system, and wide integration with electronic medical records and other already available apps and e-tools. Participants stated that to make the MHeC-C appropriate to the (Colombian) context, it needed to operate in alliance with academic institutions, health providers (at all levels), and other community organizations. Owing to the unique Colombian context, the self-report assessment needed to include items evaluating (including the creation of specific algorithms) the social determinants of health, attitudes toward and experiences with violence, and resilience, and extending the trauma module to assess childhood adverse experiences. In relation to the future build of the MHeC-C, it needed to include refinements to the interface, such as changing the color palette, designing a logo that refers to mental health, and making further modifications in language.

Although the MHeC-S was comprehensible to our Colombian participants, many changes were requested. In agreement with other authors [87], we strongly advocate for the need to adapt HITs beyond language by considering cultural variations. The same authors suggest adapting or designing HITs to acknowledge cultural differences in 4 main dimensions: content, functionality, technology platform, and user interface [44]. However, the methodology needed to achieve this has not been conceptualized. Continuing with our previous research [31], we aimed to adapt our prototype by using a modified version of our previously established R&D approach [30] within a framework comprising 2 dimensions (language and culture). During this study, a new theme emerged, which added the missing piece of the methodology, the contextual adaptation. As a result, it was possible to obtain culturally and contextually appropriate information about what is required in terms of content and functionality, as well as preferences for the prototype’s interface and the technology platform. All of this was done in a participative, collaborative, and time-efficient manner. The approach enabled us to collect information, define the needs, and find solutions on how the MHeC-C would respond to these requirements.

To make these HITs available in other languages, cultures, and places, it is necessary to tailor them beyond just language. In other words, it is important to consider them within a culturally and contextually appropriate framework. This framework should also incorporate the use of PD methodologies that involve stakeholders and end users from the beginning in the co-design, development, and adaptation of these HITs (Figure 2). To our knowledge, this paper reports the first body of research that proposes a methodology that researchers can replicate and use to adapt HITs to a myriad of cultures and contexts. A systematic use of such methodologies would finally result in the development of evidence-based, culturally sensitive, and contextually adapted HITs that are relevant, appropriate, engaging, and usable in the short and long term.

Data show that people living in rural areas receive less mental health treatment than those residing in metropolitan areas [88]. As almost one quarter of the Colombian population lives in rural areas, the systematic adaptation process used in this study allowed us to thoroughly identify the potential specific requirements for rural populations, such as the chat functionality to support local general practitioners (including those health professionals completing their social compulsory service), a geolocation system that will help tailor helplines and services available around them, and necessary adaptations of the content of the MHeC-C’s self-report assessment to reflect rural needs. Despite the proposed benefit, it is important to consider the barriers and challenges for implementing the MHeC-C in real-world settings. Mental health and digital literacy levels are common obstacles in the implementation of HITs; it is well known that many people around the world are unable to recognize mental disorders [89,90] and that this lack of knowledge associated with stigma could prevent people from seeking help and providing treatment to those in need. These problems are a particular concern in low- and middle-income countries where health services are already limited [91].

Health professionals in this study displayed some degree of apprehensiveness in relation to the aptitudes required for, and the pertinence of, viewing an automatic display of the dashboard of results for young people. Paternalistic attitudes are no longer desirable, as they increase the asymmetry in the relationship and finally lead individuals to agree with the health professional’s decisions [92,93]. The patient-centered approach and shared decision making encouraged by the MHeC-C give individuals more control and promote mutual participation, and research has shown that this type of care translates to better health outcomes and more efficient health care [94,95]. Increasing the individuals’ power, strengthening critical thinking, and empowering more informed and autonomous decisions are key concepts in HITs, as they act as digital companions by providing individuals with greater participation in the decision-making process [96]. HITs also assist health professionals in presenting their advice in a respectful manner that includes the individual’s singularity and complexity [97]. The proposed elements (dashboard of results and personalized well-being plan) of the MHeC-C could enhance the young people’s understanding of their health status, assist them in the decision-making process, build their sense of agency, and promote their functional empowerment.

Another challenge would be the integration of the MHeC-C with the current Colombian health care and benefit schedule, which is under the administration of several public and private institutions that use regulated government funds [98]. As there are many institutions that are involved in the provision of services, the MHeC-C would need to integrate with all of them to avoid perpetuating health inequities. The final goal of developing HITs is to actually develop a prototype that has great value for all end users even if the set of functionalities is different. For example, a young person would use the MHeC-C to improve their health and well-being, track their progress, and stay connected with their health professionals, whereas health professionals would use the system to inform their day-to-day practice, access support and training, and facilitate communication with those under their care. By building an appealing, usable prototype that responds to these specific needs based on end-user type, we aim to surpass the attrition law and sustain usage over time.

Our strategic partnerships made it possible for a native Colombian team of researchers to conduct all the phases (including data collection and analysis) in the Spanish language. This approach reduced the risk of losing information (or meaning) and increased research efficiency by decreasing time and costs [99]. In addition, through working closely with end users, the adapted R&D cycle allowed constant iterations of the MHeC-C in response to technological advances and end-user needs. Effective engagement with local stakeholders, use of local capacities and systems, and measurement of relevant results for the community have been identified as strategies to promote translational research in low- and middle-income countries [100].

### Implications

Countries such as Colombia, which have limited resources allocated to health (7% of its GPD), struggle to make decisions regarding where to invest to have the best outcomes. HITs show promise in reducing costs and being cost-effective in the long run [101,102]; however, the development (from conception to implementation and sustainability) is an expensive and arduous process [103,104]. At the same time, building capacity by training health professionals and increasing infrastructure is also a slow and expensive pathway [105-107]. As a solution, we proposed a rigorous methodology to adapt already available (and evidence based) HITs along 3 main pillars: language, culture, and context. A systematic use of this approach has the potential to reduce costs and to increase the number of HITs available (in different languages and cultures) in a time-efficient manner. HITs that show value in terms of content and appropriateness to context could integrate with already available health systems and finally help to breach not only physical but also technological and social health inequalities [108], making health care more accessible, affordable, and available.

The Colombian context is complex, as despite economic growth, it continues to be one of the most unequal countries in the world [109]. One quarter of its population lives in rural settings, with a low number of health professionals and limited infrastructure [10] and high levels of violence following five decades of internal conflict. This results in a high level of challenge for individuals, health professionals, health providers, and decision makers to change the delivery model as well as treatment standards. Web-based solutions mark a paradigm shift beyond the traditional models of health care delivery. Integrating physical resources with HITs would capitalize on Colombia’s heavy investment in telecommunications and could enable the Colombian population to access new resources; make better use of expertise; and provide better access for individuals, peers, and families. This should be done through collaborative interdisciplinary work with ongoing international support to capitalize on global medical knowledge and find new solutions, leading to quicker innovations in health service delivery.

### Limitations and Future Research

Although the importance of appropriately adapting HITs to the local context cannot be overstated, it must also be acknowledged that the contexts are constantly changing. For example, Colombia’s population makeup has changed since 2015 (when the workshops were conducted) because of recent migration from Venezuela. In the past year, more than 350,000 people have migrated from Venezuela [1], and at the beginning of 2019, it was calculated that there were more than 1 million Venezuelans residing in the country. Migrant populations have been identified to be at a greater risk of psychological distress or common mental disorders, and host countries must effectively respond to this. A pressing future need of the MHeC-C would be to include migrant populations; therefore, a new cycle of adaptation would be required. As an initial proposal and capitalizing on our previous research [31], the new version of the MHeC-C would include the cultural adaptation and adjustment disorder (available from the MHeC-S) items as the addition of the assessment of other risk factors, such as conditions of the migration process, level of acculturation, family reunification, perceived discrimination, and the length of time of residence in the host country [110].

Another limitation was the relatively small sample size, although this number still enabled us to collect sufficient information for an analysis in the framework and reach a saturation point. It is important to consider the large percentage of young people in Colombia and their diversity; consequently, these results cannot be extrapolated to the general population; therefore, further research is needed for tailoring the MHeC-C to rural and diverse populations. Additional research is also needed to develop the MHeC-C and test its engagement, efficacy, and effectiveness in real-world settings and engage other stakeholders, such as administration and management, peers, nongovernmental organizations, other community organizations, and senior health professionals with diverse degrees of technology literacy.

### Conclusions

In low- and middle-income countries, the potential to utilize already developed HITs for improved access to and better quality of mental health services is enormous. This would result not only in better mental health outcomes for young people but also more efficient, effective, and appropriate use of scarce health professional knowledge and clinical skills, as well as quality improvements in mental health service delivery. In this study, an adapted R&D cycle resulted in a technology solution acceptable for use by Colombian young people (and their supportive others) experiencing mental health problems as well as health professionals delivering care. This methodology should now be applied to other HITs as a means to bridge the digital and health care gaps not only in Colombia and the developing world but also globally to other communities or settings where resources are scarce, culture matters, and/or geography presents a challenge.

## Appendix

Multimedia Appendix 1 Original quotes in Spanish.

## Abbreviations

BMC: Brain and Mind Centre
CD-RISC: Connor-Davidson Resilience Scale
GDP: gross domestic product
HIT: health information technology
MHeC: Mental Health eClinic
MHeC-C: Colombian version of the Mental Health eClinic
MHeC-S: Spanish version of the Mental Health eClinic
NMHS: National Mental Health Survey
PD: participatory design
R&D: research and development
