# Supplementary material for: Involving End Users in Adapting a Spanish Version of a Web-Based Mental Health Clinic for Young People in Colombia: Exploratory Study Using Participatory Design Methodologies
Source: JMIR Ment Health. 2020 Feb 6;7(2):e15914. doi: 10.2196/15914 (PMC7055810; doi:10.2196/15914)
Supplement: Multimedia Appendix 1 [file mental_v7i2e15914_app1.docx]

## Appendix 1

Quote A: *“…the general emergency line is the same 123, but the psychological emergency line changes, for example in Bogota it is 106 and in Cartagena it is 125…”:*

La línea general de emergencias es 123, pero las líneas de atención psicológica cambia por ejemplo en Bogotá es 106 pero en Cartagena es 125

Quote B:*“…it would be very useful to geolocate the person, this means the prototype would be able to know where they are so they don’t have to waste time filling their addresses. Also, as Colombia is so diverse, we know that the regions have different needs so the questions could be specific to those needs. For example, in regions affected with violence, assessing this topic in-depth would be crucial. Another example would be assessing thoroughly the social determinants of health if the person lives in a poor area or is identified with a low socioeconomic status…”:*

Sería muy útil geolocalizar a la persona. Esto nos ayudaría a que el sistema supiera donde se encuentran y así los pacientes no tienen que perder tiempo llenado donde viven. Además, cómo vivimos en un país tan diverso en sus necesidades sería una buena idea que las preguntas reflejaran esto. Por ejemplo, en las regiones más afectadas por la violencia pudiéramos evaluar este tema en profundidad. Otro ejemplo sería en regiones más pobres o de bajo estrato socioeconómico valorar bien los determinantes de la salud…”

Quote C: *“…I would like to know more who I’m going to see, so I can decide if I see a man or a woman or see what are their areas of expertise…”:*

Me gustaría saber quien me va a atender, elegir entre un hombre o una mujer o saber quien es experto en las áreas que me interesan.

Quote D: *“…doctors in their social compulsory service (located in rural areas) might need support from specialists, it would be very useful to use the video visit system to help them assessing difficult cases or to provide supervision…”:*

Los rurales pueden necesitar ayuda de los especialistas con los casos difíciles, el Sistema de videoconferencia seria útil n estos casos para ayudarles con las valoraciones o supervisarlos

Quote E: *“…It reminds me of orange uniforms of the Colombian Civil Defense…”:*

Me hace pensar en los uniformes anaranjados de la Defensa Civil Colombiana

Quote F: *“…I might be wrong but the logo needed to include a brain or a head or something like that…”:*

Puede que esté equivocado pero creo que el logo debería incluir un cerebro o una cabeza, algo así.

Quote G: *“the country is so diverse that there are regions that use formal pronouns and others informal pronouns, the most important thing is to use it consistently…”:*

El país es tan diverso, que hay regiones que usan los pronombres formales y otras los informales, creo que lo más importante es ser consistente.
